# Effectiveness of physical barriers to prevent suicide by jumping from high-risk bridges: From an integrative review to a northern Italian province’s paradigm

**DOI:** 10.1016/j.pmedr.2024.102745

**Published:** 2024-04-27

**Authors:** Roberto Merli, Alessandra Costanza

**Affiliations:** a Department of Mental Health, Mental Health and Suicide Prevention Center, Biella, Italy; b Nel Chiostro, Medical Study Center, Vercelli, Italy; c Department of Psychiatry, Adult Psychiatry Service, Geneva University Hospitals (HUG), Geneva, Switzerland; d Department of Psychiatry, Faculty of Biomedical Sciences, University of Italian Switzerland (USI), Lugano, Switzerland; e Department of Psychiatry, Faculty of Medicine, University of Geneva (UNIGE), Geneva, Switzerland

**Keywords:** Suicide, Suicidal behavior, Suicide attempt, Suicide by jumping, High-risk location, High-lethality, Hotspot, Covid-19, Economic crisis

## Abstract

**Background:**

Although uncommon, suicide by jumping is almost always lethal and can be significantly elevated locally due to the availability of tall structures including bridges and other high-risk locations. Empirical evidence suggests that restricting access to certain suicide methods is highly effective at preventing suicide, prompting the construction of physical barriers in many high-risk jumping locations. However, some have argued that these measures are too costly and only lead to method or location substitution.

**Objectives:**

To examine whether physical barriers are effective at preventing suicide by jumping or whether method or location substitution occur.

**Methods:**

An integrative review of the most representative literature.

**Results:**

Results clearly show that physical barriers are highly effective at preventing suicide by jumping with little to no method or location substitution occurring. Furthermore, their cost is far outweighed by the monetary benefits of averted suicides.

**Conclusions:**

Using the Italian pre-alpine/alpine areas as a paradigm, we argue that local topography and socioeconomic particularities led to significantly elevated rates of suicide in general, and of suicide by jumping from bridges in particular, especially among young adults who are more vulnerable to economic crises and show elevated susceptibility to impulsive acts, a known characteristic associated with suicide by jumping, which makes the issue even more topical and clinically relevant. As these finding are easily generalized to other territories with similar topographical and/or economic characteristics, we end with a call to action, urging local authorities to heed the scientific evidence and take the necessary steps to improve suicide prevention.

## 1 Introduction

While jumping from heights is a relatively uncommon means of suicide, it is almost always lethal (Bennewith et al., 2011). On average, it accounts for less than 10 % of suicides except in areas with easy access to tall bridges or buildings where this percentage can be much higher (e.g., New York City: 24 %, Hong Kong: 45 %, Singapore: 60 %, and San Francisco: 70 %) (Beautrais, 2007, Beautrais et al., 2010, Blaustein and Fleming, 2009, Gross et al., 2007, Gunnell and Nowers, 1997, Wong et al., 2014). While sites that attract a large number of suicides were previously referred to as “hotspots” or “magnets”, these terms are now considered inappropriate as they trivialise the act and advertise the location as “good” for suicide. We will therefore use the terms “high-risk” or “frequently-used location” instead when referring to a place where suicides occur with a disproportionate frequency and create identifiable clusters. Through a vicious circle, this notoriety may contribute to further increase the appeal and confer a unique meaning to these places for individuals who are in suicidal crisis (Owens, 2016, Reisch, 2009, Reisch and Michel, 2005, Reisch et al., 2006, Waalen et al., 2020). High-risk locations can include not only bridges and skyscrapers, but also viaducts, monuments, and places found in nature, such as waterfalls, steep rocks, or cliff tops. Furthermore, these places may be highly transient, i.e., they emerge quickly and are rapidly replaced by another location (Owens et al., 2006), prompting Reisch and colleagues to propose defining a minimum period during which an excess of suicides (>0.4 suicides per year over a 10-year period) needs to occur as a criterion for labelling a site as “high-risk” (Reisch and Michel, 2005, Reisch et al., 2006).

There is overwhelming evidence that restricting access to a certain suicide method is one of the most effective suicide-prevention measures (Bertolote, 2004, Hawton, 2007, Kim et al., 2019, Lin et al., 2021, Mann et al., 2005, Yip et al., 2012, Zalsman et al., 2016). In the context of suicide by jumping, this led to many authors pointing out the benefits of securing high-risk locations with physical barriers and fences (Beautrais, 2007, Beautrais, 2007, Cox et al., 2013, Larsen et al., 2020, Pirkis et al., 2013, Pirkis et al., 2015). The main arguments brought forward in debates surrounding the construction of physical barriers typically revolve around evidence-based practices (supposed lack in evidence that barriers are effective), cost-effectiveness (barriers are overly costly), aesthetic concerns (landmark bridges and their picturesque views could be marred by barriers), method and location substitution (installing barriers on a particular bridge will only lead suicidal individuals to seek other methods or locations), and related personal opinions and agendas (Pirkis et al., 2013, Atkins Whitmer and Woods, 2013, Bandara et al., 2022, Beautrais et al., 2009, Sinyor et al., 2017).

In this paper, we review the current scientific knowledge on the effectiveness of physical barriers for preventing suicide by jumping before directing our focus to the pre-alpine and alpine arc areas of northern Italy, where topographical and socioeconomic particularities led to significantly elevated rates of suicide in general and of suicide by jumping in particular.

This integrative review aims to provide a synthesis of the current knowledge and its applicability to practice, to encourage policy actions that prevent suicide by this method in any area characterized by “high-risk” bridges or heights. The pre-alpine and alpine arc areas of a northern Italian province are used as a paradigm, also in the context also of a growing uncertainty about the post COVID-19 economic situation particularly among youth.

### 1.1 Factors attracting individuals to specific locations to suicide by jumping

Certain places or structures hold a certain renown with the public or are of personal significance to some individuals, factors that may come into play during a suicidal crisis (Ellis, 1996, Rosen, 1975, Sinyor and Levitt, 2010). For instance, survivors of attempted suicides at a bridge in San Francisco, USA, stated that the bridge was iconic and possessed a special meaning for them which made them choose that particular location (Blaustein and Fleming, 2009, Rosen, 1975). Some even indicated that if it had not been possible for them to jump from their chosen location, they would not have considered changing to another location or method (Rosen, 1975). Other suicide attempters stated that they were drawn to a particular bridge by its easy accessibility, romantic location for dying, architectural beauty, and popularity in the press (Blaustein and Fleming, 2009).

Factors that affect the attractiveness of a location include easy accessibility, lethality (height) of a jump, and a unique, scenic location (Sinyor and Levitt, 2010). The strong appeal of high-risk locations means that individuals in suicidal crisis sometimes travel there from afar to take their own lives (Lam et al., 2017), a phenomenon that has been termed somewhat nonchalantly as “suicide tourism” (Gross et al., 2007, Zhi et al., 2019).

### 1.2 The role of encountering an obstacle in suicide by jumping

Jumping from a height is a means that lends itself very well to suicides with a high acute component of impulsivity since it does not require complex planning (Cheah et al., 2008). Individuals rescued after an unsuccessful suicide attempt from a high-risk bridge in San Francisco, USA, stated that they were grateful that the acute moment of crisis during which they were determined to jump had passed and they were saved; some regretted the attempt and a large majority did not wish to try again (Rosen, 1975, Seiden, 1978). In these situations, barriers can act as an effective deterrent, because they represent an obstacle to suicide attempters that may allow the acute moment to pass. However, the so-called “impulsive” suicide still lacks a proper definition and cannot simply be attributed to generic impulsivity (May and Klonsky, 2016, Swann et al., 2020). The role played by generic impulsivity and its link to suicide is increasingly being discussed in the literature, particularly in the context of potential relationships with other associated factors such as aggression and anger traits, the so-called impulsivity-endophenotypes (Brent et al., 1994, Giegling et al., 2009, Turecki and Brent, 2016). This appears particularly relevant in suicidal population subgroups such as youths, who, for neurobiological reasons, often exhibit a physiological imbalance between the maturation of the frontal lobes versus the amygdala (Costanza et al., 2021). Of note, a Swiss study found that individuals who suicide by jumping from bridges were on average 14.3 years younger than those who suicide by jumping from other sites and 10 years younger than those who suicide by other methods (Reisch et al., 2008).

## 2 Methods

Several evidence-based studies have investigated the effectiveness of physical barriers for preventing suicide by jumping. We performed an integrative review by searching in four major electronic databases comprising medical and social science research (PubMed/MEDLINE, Scopus, Science Direct, and PsychINFO) for relevant studies between January 1990 and August 2023. Only studies published in English have been considered. The review explores the issue from four different angles: Jumping from high-risk bridges, jumping from other high-risk locations such as places found in nature and other buildings, the cost-effectiveness of barriers, and findings by previous systematic reviews and *meta*-analyses. Because of this structure, and to avoid redundancy, we present the results and their discussion in the same section. The most relevant works are reviewed in chronological order (from oldest to most recent).

## 3 Results and discussion

### 3.1 Jumping from high-risk bridges

In the early 1990 s Lester (Lester, 1993) and O’Carroll and Silverman (O'Carroll and Silverman, 1994) investigated the effectiveness of barriers at a high-risk bridge in Washington DC, USA, finding that a 2.5 m barrier was able to reduce the rate of suicide by jumping to zero within one year without any statistically significant increase at a neighboring location. Beautrais (Beautrais, 2001) found that suicides increased five-fold after removing a medium-height barrier from a high-risk, inner-city bridge in Auckland, New Zealand. When the local council, in response to this sudden increase in suicides, erected a new, taller barrier, the rate of suicides by jumping dropped to zero (Beautrais et al., 2009). In 2007, Bennewith et al. (Bennewith et al., 2007) compared suicide rates during the 5 years before to the 4 years after installing a 2 m barrier and found that suicide rates were halved post-barrier without any measurable displacement to other locations. From interviews with bridge staff, they concluded that even if suicidal individuals are able to climb the physical barrier, this sometimes delayed them sufficiently to allow human interventions and prevent the individual from jumping (Bennewith et al., 2011). At the same time, having investigated suicides by jumping during the 45-year period from 1960 to 2005 at a high-risk bridge in Maine, USA, where a 3 m safety fence had been installed in 1983, Pelletier found that no new suicides by jumping had been recorded at this location in the 22 years post-barrier while suicides by jumping did not show any increase at other locations (Pelletier, 2007). In a Swiss national survey comparing suicide rates for the period from 1990 to 2003 in regions with and without suicide bridges to estimate the effects of protective interventions on method and site substitution, Reisch and colleagues (Reisch et al., 2007) found that only about one third of individuals would jump from buildings or other structures if no bridge was available. While they did not find any method substitution in women, men tended to substitute jumping by overdosing in regions without suicide bridges. However, their overall conclusion was that restricting access to bridges did not lead to method or site substitution and that securing bridges with protective barriers would result in lives saved. Sinyor et al. (Sinyor et al., 2017, Sinyor and Levitt, 2010) conducted a similar analysis at a high-risk bridge in Toronto, Canada, and found that the installation of a 5 m tall barrier led to a decline from 9.0 to 0.1 suicides per year at this particular location. In their initial study, published four years after the barrier had been constructed (Sinyor and Levitt, 2010), they concluded that overall suicide rates by jumping remained unchanged in Toronto owing to a statistically significant increase in suicides at other bridges. However, upon revisiting the questions seven years later (Sinyor et al., 2017), they no longer found any measurable increase in suicides at other Toronto bridge locations. Commensurate with findings by Law et al. (Law et al., 2014) (see below), they stressed that research investigating the effectiveness of barriers should interpret short-term results with caution as displacements to other bridges may occur immediately post-barrier (in their case, they attributed this to a media effect), while in the long term, the installed barriers led to a significant, city-wide reduction in suicides by jumping. Perron and colleagues (Perron et al., 2013) investigated whether the installation of a 2.5 m suicide prevention barrier on a high-risk bridge in Montreal, Canada, led to a reduction in suicide rates and/or a displacement to other jumping sites nearby. Compared to the 14-year period preceding the installation of the barrier, the incidence rate dropped by 76 % during the 4 years immediately following the barrier installation, with little to no displacement to other structures and jump sites, thus leading to an overall decrease in suicides by jumping across the entire province.

Law et al. (Law et al., 2014) found a similar result for a high-risk bridge in Brisbane, Australia, where the installation of a barrier led to a reduction in suicide rate of about 53 % in the 3 years immediately post-barrier, which increased to 96 % in the subsequent 3 years, and eventually reached 100 % after 17 years, yielding a time-averaged reduction of 87.5 %. They did not find any displacement to other locations in the same city. However, while the city-wide rate of suicide by jumping decreased by 32.4 %, the overall suicide rate in Brisbane remained unchanged. In a more comprehensive study that included 15 high-risk locations across Switzerland, Hemmer and coworkers (Hemmer et al., 2017) found that physical barriers reduced suicide rates by 69 % on average, stressing that a barrier’s effectiveness greatly depended on its height and overall coverage of the structure. Only barriers with a minimum height of 2.3 m and covering the entire length of the bridge were fully effective at reducing suicide rates. Another national survey was conducted in Norway by Sæheim et al. (Sæheim et al., 2017), involving 71 cases of jumping from bridges between 1999 and 2010, 33 of which had occurred at only six bridge sites. During the study period, two of these six bridges were equipped with full-length barriers and one with a barrier along the main section only. While the bridges with full-length barriers went from 11 suicides pre-barrier to zero suicides post-barrier, jumps continued from the unsecured parts of the bridge that had only been partially secured.

In a study examining the effect of a 2.4 m suicide fence installed on an inner-city, high-risk suicide bridge in Washington DC, USA, Berman and colleagues (Berman et al., 2022) examined a 37-year dataset, comparing 7 years of pre-barrier with 30 years of post-barrier suicide data. They found that suicide rates dropped from 2.83 per year pre-barrier to 0.13 post-barrier (95 % reduction) and observed only a short-lived (for 1 year post-barrier) displacement to other bridges in the city, which disappeared in subsequent years.

### 3.2 Jumping from other high-risk locations: Places found in nature and other buildings

Other studies have focused on the effectiveness of protective structures at other high-risk locations such as cliff tops (Lockley et al., 2014, Ross et al., 2020) and high (iconic) buildings (Reisch and Michel, 2005). For instance, Lockley et al. (Lockley et al., 2014) conducted a case study at a high-risk cliff site near Sidney, Australia, where a relatively low 1.3 m barrier led to a measurable (albeit non-statistically-significant) reduction in suicides by jumping, partly due to an increase in police call-outs leading to interventions before the suicidal individual had reached the cliff’s edge. Ross et al. (Ross et al., 2020) re-examined the data from the same cliff site, compared pre- and post-intervention suicide numbers for a wider geographical area, and considered the combined effect of the entire range of suicide prevention methods that had been installed, namely the aforementioned 1.3 m fence along the cliff top, CCTV surveillance, police protocols for responding to suspicious behavior, as well as phone booths and signs displaying the number of a local suicide help line. While there was a significant increase in suicides during the 10-year period leading up to the installation of these interventions, they found a significant downward trend in female but not in male jumping suicides once the measures were in place; in fact, male jumping suicides increased slightly. They concluded that due to its low height, the 1.3 m barrier clearly did not represent a serious physical obstacle but may have served as a psychological barrier, especially for women.

### 3.3 The cost-effectiveness of physical barriers

Some authors also investigated the economic aspects related to the construction of suicide barriers, as cost arguments are often brought forward against their construction. For instance, Atkins Whitmer et al. (Atkins Whitmer and Woods, 2013) investigated a high-risk bridge in San Francisco, USA, where over 30 suicides by jumping occur each year. They compared the estimated cost of a proposed barrier (construction plus maintenance for 20 years) to the estimated reductions in mortality and the associated monetary benefit, which they quantified using estimates of the so-called “value of statistical life”, a measure typically used to attribute a monetary value to human life in road accidents by accounting for losses in wages or salary due to injury or death. For their study, they assumed that all suicides prevented by the barrier would reattempt suicide with alternative methods; the estimated reduction in mortality is thus simply due to differences in lethality between suicide by jumping (∼100 %) and the alternative methods (∼47 %). Based on this approach, they found that the barrier was a highly cost-effective means for reducing suicide mortality, leading to cost savings of the order of hundreds of millions of US$ over the 20-year period. Considering that most of the studies reviewed here (see above) did not find any significant substitution effects, neither in method nor location, this estimate would seem rather conservative as the reduction in mortality is likely higher. A similar result was obtained by Bandara and colleagues (Bandara et al., 2022) who investigated the cost-effectiveness of suicide barriers at bridge and cliff sites. They used an economic modeling approach to estimate the costs, monetary benefits, and reductions in mortality if barriers were installed at 26 easily accessible bridge and cliff sites across Australia that had reported suicides by jumping. Using return-on-investment as their primary outcome, they found that for every dollar invested, the return during the first 10 years would be 2.4 dollars with total savings amounting to almost 300 million US$. They concluded that physical barriers were a highly cost-effective suicide prevention intervention.

### 3.4 Findings by systematic reviews and *meta*-analyses

Pirkis and colleagues (Pirkis et al., 2013) conducted a *meta*-analysis involving nine studies to assess the effectiveness of physical barriers (some of which have been included here individually due to their historic relevance and significance as important landmark studies). They found that, on average, barriers led to a 86 % reduction in jumping suicides per year, with an associated 44 % increase at nearby sites, yielding a net reduction of 28 % across all sites. However, it should be noted that these findings rely to a large degree on studies whose absolute SA numbers were rather low, such that the corresponding 95 % CI makes a reduction in suicides at nearby locations almost as likely as an increase (see Figure 2B in (Pirkis et al., 2013). Among the two studies with higher SA numbers (and thus more robust statistics) was Sinyor and Levitt (Sinyor and Levitt, 2010) who found a 63 % increase at nearby bridge locations in the years immediately following barrier construction. However, in a later study by the same authors (Sinyor et al., 2017), they state that this increase was short-lived and likely due to some unfortunate media reports about bridge suicides at the time. Importantly, the long-term trend no longer showed a 63 % increase but a 20 % decrease at nearby bridge locations. Hence, if this data had been available to Pirkis et al. (Pirkis et al., 2013), the resulting overall percentages would change significantly.

In a second review and *meta*-analysis, they compared various intervention methods such as barriers/safety nets (restricting access to means), signs displaying emergency help line numbers (encouraging help seeking), and CCTV surveillance (increasing the likelihood of third-party interventions) and found that restricting access to means (e.g., through physical barriers) was far more effective (incidence rate ratio of 0.09) than to the other two approaches (both with incidence rate ratios of approx. 0.5) (Pirkis et al., 2015).

In summary, and as confirmed by a recent Cochrane review (Okolie et al., 2020), there is overwhelming evidence that physical barriers are highly effective at reducing suicides by jumping without any long-term displacement to other structures or substitution by other methods. If the barriers are sufficiently tall and cover the entire length of the bridge, the reduction is typically close to or exactly 100 %. In addition, the construction of barriers is cost-effective as the cost of installation and maintenance is far outweighed by the monetary savings associated with the reduction in mortality. Table 1 provides a summary of the literature on suicide by jumping from high-risk bridges reviewed in this study and their main findings. It is focused on high-risk bridges which provide a good example of how scientific data can be applied to illustrate practical concerns and applications such as the usefulness of physical barriers at such sites.

**Table 1:** Main studies (in chronological order) examining the effectiveness of physical barriers on high-risk bridges for preventing suicide by jumping. By A-B study design we refer to a two-phase study comparing the post-intervention period B to the pre-intervention baseline A.

**Author(s)**	**Country**	**Type of Study**	**Main results**
Lester (Lester, 1993)*	USA	Before-and-after single site study (A-B design)	Installing barriers significantly decreased the number of suicides by jumping without any increase at adjacent bridge locations.
O’Carroll et al. (O'Carroll and Silverman, 1994)*	USA	Before-and-after single site study (A-B design)	Installing 2.5 m tall barriers decreased the number of suicides by jumping to zero without any increase at other locations.
Beautrais et al. (Beautrais et al., 2009, Beautrais, 2001)*	New Zealand	Before-and-after single site study (A-B-A reversal design)	Removal of barriers was followed by a 5-fold increase in suicides. After reinstalling full-length safety barriers no more suicides by jumping were recorded.
Bennewith et al. (Bennewith et al., 2007)*	UK	Before-and-after single site study (A-B design)	Installing 2 m tall barriers along parts of the bridge halved the number of deaths by jumping during the following 4 years without any displacement to other locations.
Pelletier (Pelletier, 2007)*	USA	Before-and-after single site study (A-B design)	With a tall (3 m) safety fence, the number of suicides dropped from 14 (during 23 years pre-barrier) to zero (during 22 years post-barrier) without any increase at other locations.
Reisch et al. (Reisch et al., 2007)	Switzerland	Before-and-after multi-site study (A-B design)	Investigating the effects of barriers on method and site substitution, they found no substitution in women and some method substitution in men in those regions where no other suitable bridge was available.
Sinyor et al. (Sinyor et al., 2017, Sinyor and Levitt, 2010)	Canada	Before-and-after single site study (A-B design)	Suicide rates declined from 9.0 deaths/year pre-barrier to 0.1 post-barrier (5 m) with a short-lived increase at nearby sites and no associated increase in suicide by other means.
Atkins Whitmer et al. (Atkins Whitmer and Woods, 2013)	USA	Cost effectiveness single site study	Installing barriers would save lives and be highly cost effective.
Pirkis et al. (Pirkis et al., 2013, Pirkis et al., 2015)**	n/a	Systematic review and *meta*-analysis of data from 9 studies	Barriers resulted in an 86 % reduction in suicides by jumping with a 44 % increase at nearby sites, yielding a net decrease by 28 %. Barriers are much more effective compared to other interventions that encourage help seeking or third-party intervention.
Perron et al. (Perron et al., 2013)	Canada	Before-and-after single site study (A-B design)	Suicide rates by jumping decreased by over 75 % after installing a 2.5 m barrier with little to no displacement to other sites.
Law et al. (Law et al., 2014)	Australia	Before-and-after single site study (A-B design)	Tall (3.3 m) barriers reduced the number of suicides by 53.0 % (p = 0.041) immediately after installation and by 100 % in subsequent years, with no evidence of displacement to other bridge locations.
Hemmer et al. (Hemmer et al., 2017)	Switzerland	Before-and-after multi-site study (A-B design)	On average, barriers reduced suicides by about 69 %, with reductions of 100 % recorded for taller barriers (2.3 m minimum) that cover the entire length of the bridge.
Sæheim et al. (Sæheim et al., 2017)	Norway	Before-and-after multi-site study (A-B design)	Full-length barriers led to a 100 % reduction in suicides by jumping at 2 bridges, while suicides continued at a bridge that was only partially secured.
Berman et al. (Berman et al., 2022)	USA	Before-and-after multi-site study (A-B design)	Installation of a 2.4 m suicide fence at a high-risk inner-city bridge reduced suicides by 95 % without any lasting increase at other bridges in the same city.
Bandara et al. (Bandara et al., 2022)	Australia	Modeling study of cost effectiveness	Installing barriers at bridge and cliff sites would yield a 240 % return on investment over 10 years and produce significant monetary savings.

*These studies are covered by the *meta*-analysis by Pirkis et al. (Pirkis et al., 2013) and have only included as separate items due to their historic relevance and significance as important landmark studies.

**The percentages reported in the Main result column are for Pirkis et al. (Pirkis et al., 2013). The 44 % increase at nearby sites is largely due to findings be Sinyor and Levitt (Sinyor and Levitt, 2010) who found a 63 % increase at nearby bridge locations. However, in a later study by Sinyor et al. (Sinyor et al., 2017), published 4 years after Pirkis et al., the authors found an overall decrease by 20 % at nearby locations and attributed the initial increase of 63 % to unfortunate media coverage immediately following the installation of the barrier.

## 4 Toward practice

### 4.1 Lack of physical bridge barriers in northern Italy

**Fig. 1:**
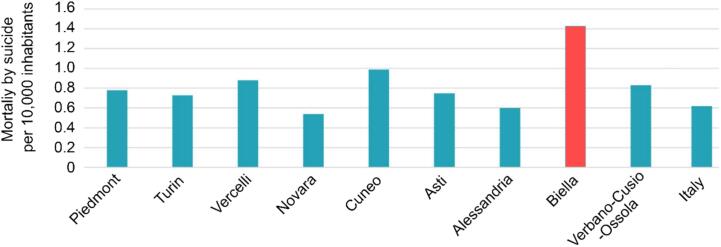
Mortality by suicide in 2019 for the Piedmont region, various Piedmont provinces, and Italy.

Sources: Coggiola et al., 2020, ISTAT, 2019, Suicide, 2019

The pre-alpine and alpine arc areas of northern Italy are mountainous regions with numerous high-risk bridges, many of which lack appropriate physical barriers. It has been hypothesized that the mere availability of high bridges in a certain area can lead to regionally elevated rates of suicide by jumping (Värnik et al., 2009). Other factors have been linked to high-lethality suicide attempts such as youth or pollution, impulsivity, and environmental conditions which appear to be linked to male gender (Aguglia et al., 2021, Amerio et al., 2021). This is exemplified by Biella, a Piedmont province, with a suicide rate that is consistently above the regional and national averages. For instance, the 2019 suicide rate in Biella exceeded both the Piedmont regional and Italian national averages by 85 % and 112 %, respectively (Fig. 1) (Coggiola et al., 2020, ISTAT, 2019, Suicide, 2019). During the first decade of this century, Biella suicide rates were up to three times higher than the national average (Table 2) (ISTAT, 2019). While different risk factors may combine to explain these locally elevated suicide rates, two particular factors stand out: (i) the mountainous terrain with many high-risk bridges available as potential jump sites and (ii) the severe repercussions of the 2008 economic crisis which may be mirrored by the current economic downturn related to Covid-19 aftermaths.

**Table 2:** Suicide rates (per 100,000 inhabitants) from 2005 to 2010 for Italy, Piedmont, and Biella.

	**2005**	**2006**	**2007**	**2008**	**2009**	**2010**
**Biella**	8.5	14.9	12.3	11.2	11.7	9.6
**Piedmont**	7.4	7.5	7.5	7.6	5.9	5.3
**Italy**	4.9	5.2	4.8	4.7	5.0	5.1

### 4.2 The example of Biella’s high-risk bridges

In the Biella province, there are two bridges that account for 80 % of all suicides by jumping (Merli and Etzersdorfer, 2015) and both meet several of the criteria outlined above for high-risk locations. The first bridge is easily accessible for pedestrians, its height of 152 m guarantees the lethality of the jump, and its press coverage as an internationally renowned location for bungee jumping means that it is well known throughout northern Italy and beyond. Although it currently has a 2 m barrier installed, this height has been shown to be insufficient for deterring jumpers (Hemmer et al., 2017) and its structural particularities make it easy to climb. Of note, the proportion of “suicide tourism” (i.e., the number of suicides by non-Biella residents) at this location increased from 8.3 % during 1994–2003 to 41.7 % between 2004–2013 (unpublished internal data), possibly due to the growing notoriety of this bridge as a suicide location.

The second bridge was built in the 1960 s with a length of 605 m and a height of 33 m and also has easy pedestrian access with a railing that is merely 1 m high. It is less well known and therefore only attracted one out-of-town suicide during the 1994–2013 period, while the remaining suicides were by residents of Biella province (Merli and Etzersdorfer, 2015). In 2019, gates were installed to limit pedestrian access, followed by cameras/alarms connected to optical sensors that can alert a surveillance service, and finally the height of the railing was increased through added netting. While these modifications are too recent to attempt any meaningful assessment regarding their effectiveness (see the findings regarding short-term displacement effects mentioned above), future studies should compare post-intervention suicide rates at this bridge to those at the first bridge (which only has insufficient protection) to provide an objective measure of the effectiveness of these new barriers and other protection measures.

In contrast, a 47 m tall bridge that was built in 1886 and retrofitted between 1900 and 1910 with a 2.30 m high security barrier, presumably in response to several suicides by jumping at the time, only had two suicides since 1994. In addition, the few suicides that occurred at this location did not receive much media attention, which prevented this bridge from gaining notoriety, often a prerequisite to trigger imitation suicides (Merli and Etzersdorfer, 2015, Niederkrotenthaler et al., 2010, Sonneck et al., 1994).

### 4.3 Economic downturns and their impact on the suicide rate in Biella

Biella province had an important and flourishing textile industry. Starting in 2001, this sector saw an initially insidious but increasingly dramatic decline with unemployment rates more than tripling by 2008 as a result of the economic downturn, a well-known risk factor for suicide, especially in men (Chang et al., 2013, Economou et al., 2016, Economou et al., 2013, Pompili et al., 2014, Rachiotis et al., 2015). The suicide rate had already increased years before the peak of the economic crisis in 2008 (in 2005 it was already about twice the Piedmont and three times the national averages, Table 2) (ISTAT, 2019). During the 2006–2010 economic crisis, the Piedmont suicide rate decreased, approaching the Italian national average, while the Biella rate remained at about twice the Piedmont regional and the Italian national averages (with an increase particularly among young men (Pompili et al., 2014).

Just as the 2008 economic crisis led to an increase in suicides, we expect that the current economic crisis related to aftermaths of the Covid-19 pandemic and the ongoing war in Ukraine will have similar consequences, further exacerbating the situation in an area where suicide rates are already elevated (Costanza et al., 2021). Several recent studies suggested that there might be a delayed response in suicide rates following the acute phase of the COVID-19 pandemic and the ensuing economic crisis (Ambrosetti et al., 2021, Gruber et al., 2021), possibly related to a condition of widespread demoralization (Costanza et al., 2022, Clarke and Kissane, 2002, Costanza et al., 2020) (although the war in Ukraine is too recent for any meaningful analysis of the mental health impact of the associated economic crisis). Recent publications have highlighted that youths are highly vulnerable to Covid-19 related fears and psychic suffering (Costanza et al., 2021), with some gender differences (Amerio et al., 2021), possibly also linked to the higher impulsivity associated with the male gender (Pompili et al., 2014). Others reported lasting increases in youth suicidal behavior following the acute phased of the Covid-19 pandemic (Ambrosetti et al., 2021, Dubé et al., 2021, Pompili, 2021, Sher, 2020, Sher, 2021, Varma et al., 2021, World Health Organization, 2022). In this context, the need to equip bridges in the Biella region with physical barriers is even more relevant and topical.

## 5 Limitations

The manuscript has several limitations. Firstly, it is an integrative and not a systematic review (a choice dictated by the greater potential of the former to inform healthcare policy and practice). Secondly, the manuscript employs a structure that is methodologically atypical. We expressly chose this approach to be able to move from current knowledge data toward clinical practice while avoiding unnecessary redundancy. For the same reasons, we present the results directly along with their discussion and a paradigm derived from daily clinical practice. Lastly, while integrative reviews necessarily detail work conducted in the past, the paper clearly aims to provide possible future perspectives, particularly since the issue is still ongoing and under active investigation with only small amounts of objective data having become available to date. A follow-up paper is being planned which will present a more detailed data analysis, that will go beyond longitudinal analyses over time and include a direct comparison between bridges with and without precautionary measures.

## 6 Conclusions

Suicide by jumping remains a serious global problem, particularly in locations with a ready availability and easy access to tall structures such as bridges, buildings, or cliff tops. In this viewpoint article we summarized the current knowledge regarding the effectiveness of erecting physical barriers to reduce suicide by jumping from high-risk bridges and other structures or natural places. The literature contains overwhelming evidence to show that physical barriers are highly effective at reducing suicides by jumping. While some studies found indications of method or location substitution, these were typically short-lived and still resulted in a net overall reduction in suicides by jumping. However, the large majority of studies did not find any evidence for measurable substitution of place or method. To be fully effective, barriers should be sufficiently high (at least 2.3 m) and cover the entire length of the bridge. Focusing on cost aspects, several studies have shown that physical barriers are a highly cost effective means of preventing suicides, with the economic benefits of the lives saved far outweighing the costs of barrier installation and maintenance. The second part of this article focused on the particular case of the pre-alpine and alpine arc areas of northern Italy, a mountainous region where suicide rates are consistently twice to thrice the national average. We argue that these increased suicide rates are likely associated with the availability of many high-risk bridges, most of which lacking adequate protective structures, and the particular socioeconomic situation, i.e., a surge in unemployment following the 2007/8 economic crisis. This increase in suicides disproportionally affected young adults who often have a strong impulsive thrust (a characteristic frequently associated with suicide by jumping) and who are particularly vulnerable to economic downturns (through disproportionate increases in youth unemployment). Given the strong scientific evidence of the effectiveness of physical barriers and the expected increase in suicides (from an already elevated level) in response to the current economic downturn related to the aftermaths of the COVID-19 pandemic, the critical lack of suicide barriers requires incisive and immediate action by local decision makers. As these findings are readily generalizable to other territories that are characterized by high-risk bridges or heights, we end with a call to action, urging local authorities to heed the scientific evidence and take the necessary steps to save lives. Any further delay in their implementation could be considered negligent.

## CRediT authorship contribution statement

**Roberto Merli:** Writing – original draft, Validation, Supervision, Project administration, Conceptualization. **Alessandra Costanza:** Writing – original draft, Writing – review & editing, Methodology, Conceptualization.

## Declaration of competing interest

The authors declare that they have no known competing financial interests or personal relationships that could have appeared to influence the work reported in this paper.

## Data Availability

No data was used for the research described in the article.
